# Impact of insulin-like growth factor-1 receptor and amphiregulin expression on survival in patients with stage II/III gastric cancer enrolled in the Adjuvant Chemotherapy Trial of S-1 for Gastric Cancer

**DOI:** 10.1007/s10120-016-0600-x

**Published:** 2016-02-16

**Authors:** Wataru Ichikawa, Masanori Terashima, Atsushi Ochiai, Koji Kitada, Issei Kurahashi, Shinichi Sakuramoto, Hitoshi Katai, Takeshi Sano, Hiroshi Imamura, Mitsuru Sasako

**Affiliations:** 1 Division of Medical Oncology, Showa University Fujigaoka Hospital, 1-30 Fujigaoka, Aoba-ku, Yokohama, Kanagawa 227-8501 Japan; 2 Division of Gastric Surgery, Shizuoka Cancer Center, 1007 Shimonagakubo, Nagaizumi-cho, Sunto-gun, Shizuoka, 411-8777 Japan; 3 Pathology Division, Exploratory Oncology Research and Clinical Trial Center, Research Center for Innovative Oncology, National Cancer Center Hospital East, 6-5-1 Kashiwanoha, Kashiwa, Chiba 277-8577 Japan; 4 Department of Surgery, National Hospital Organization Fukuyama Medical Center, 4-14-17 Okinogami-cho, Fukuyama, Hiroshima 720-8520 Japan; 5 Data Innovation Center, iAnalysis, Inc., 2-2-15-1403 Minamiaoyama. Minato-ku, Tokyo, 107-0062 Japan; 6 Department of Surgery, Saitama Medical University International Medical Center, 1397-1 Yamane, Hidaka, Saitama 350-1298 Japan; 7 Gastric Surgery Division, National Cancer Center Hospital, 5-1-1 Tsukiji, Chuo, Tokyo, 104-0045 Japan; 8 Department of Gastroenterological Surgery, Cancer Institute Hospital, Japanese Foundation for Cancer Research, 3-8-31 Ariake, Koto-Ku, Tokyo, 135-8550 Japan; 9 Department of Surgery, Toyonaka Municipal Hospital, 4-14-1 Shibahara-cho, Toyonaka, Osaka 560-8565 Japan; 10 Department of Surgery, Hyogo College of Medicine, 1-1 Mukogawa-cho, Nishinomiya, Hyogo 663-8501 Japan

**Keywords:** Gastric cancer, ACTS-GC study, *IGF1R*, *AREG*

## Abstract

**Background:**

Exploratory biomarker analysis was conducted to identify factors related to the outcomes of patients with stage II/III gastric cancer using data from the Adjuvant Chemotherapy Trial of S-1 for Gastric Cancer, which was a randomized controlled study comparing the administration of an orally active combination of tegafur, gimeracil, and oteracil with surgery alone.

**Methods:**

Formalin-fixed paraffin-embedded surgical specimens from 829 patients were retrospectively examined, and 63 genes were analyzed by quantitative real-time RT-PCR after TaqMan assay-based pre-amplification. Gene expression was normalized to the geometric mean of *GAPDH*, *ACTB*, and *RPLP0* as reference genes, and categorized into low and high values based on the median. The impact of gene expression on survival was analyzed using 5-year survival data. The Benjamini and Hochberg procedure was used to control the false discovery rate.

**Results:**

*IGF1R* and *AREG* were most strongly correlated with overall survival, which was significantly worse in high *IGF1R* patients than low *IGF1R* patients, but better in high *AREG* patients than low *AREG* patients. The hazard ratio for death in the analysis of overall survival (S-1 vs. surgery alone) was reduced in the high *IGF1R* group compared with the low *IGF1R* group and in the low *AREG* group compared with the high *AREG* group. There were no significant interaction effects.

**Conclusion:**

*IGF1R* gene expression was associated with poor outcomes after curative resection of stage II/III gastric cancer, whereas *AREG* gene expression was associated with good outcomes. No significant interaction effect on survival was evident between S-1 treatment and gene expression.

**Electronic supplementary material:**

The online version of this article (doi:10.1007/s10120-016-0600-x) contains supplementary material, which is available to authorized users.

## Introduction

Despite a decreasing trend in Japan, gastric cancer remains the second most common cause of cancer-related death worldwide. Adequate surgery is the only treatment known to offer a cure, with adjuvant therapy improving overall survival (OS). The Adjuvant Chemotherapy Trial of S-1 for Gastric Cancer (ACTS-GC), which was a prospective randomized phase III trial, demonstrated that surgery plus treatment with an orally active combination of tegafur, gimeracil, and oteracil at a molar ratio of 1:0.4:1 (TS-1; Taiho Pharmaceutical, Tokyo, Japan) was more effective than surgery alone in Japanese patients with stage II/III gastric cancer [1–3]. The 5-year OS rate was 71.7 % in the S-1 group versus 61.1 % in the surgery-alone group. However, the 5-year overall survival (OS) rate in patients with stage IIIB disease was 50.2 % in the S-1 group in a subset analysis, suggesting room for improvement. This finding highlighted the need to identify the factors influencing relapse to develop more effective treatments for high-risk gastric cancer patients.

We have already published two papers on ACTS-GC biomarker studies. Terashima et al. tested HER2 and EGFR for their potential as markers by performing an immunohistochemical assay, and reported that EGFR is a poor prognostic marker, and not a predictive marker [4]. Sasako et al. tested genes involved in pyrimidine metabolism (*TS*, *DPD*, *OPRT*, *TP*) for their potential as predictive markers by performing an RT-PCR assay, and reported that *TS* and *DPD* mRNAs are better predictive markers [5]. The methods used in these two articles together constitute the so-called candidate approach. In the present study, we expanded the number of genes up to 63, compared with the aforementioned candidate approach that used only a few genes, to investigate a prognostic or predictive marker for S-1 therapy. We have included genes encoding key molecules such as those involved in growth factor signaling pathways, apoptotic signaling pathways, and DNA repair mechanisms, as well as 5-FU-related genes. This method is based on previous reports, which showed that the molecules involved in growth factor signaling pathways, apoptotic signaling pathways, and DNA repair mechanisms served as prognostic factors and significant predictive markers in the development of the fluorinated pyrimidine-based anticancer agent against stomach cancer [6, 7]. Thus, we could perform hypothesis-driven testing of the panel of 63 genes selected on the basis of their biological functions and relationships reported in the literature. Furthermore, in previous reports published by Sasako, a real-time RT-PCR technique without pre-amplification was used for mRNA detection. In the present study, we used a highly sensitive detection procedure involving multiplex pre-amplification of 14 cycles before real-time PCR detection with TaqMan Array Cards on FFPE samples. This procedure enabled us to detect low gene expression levels more precisely than did the previous procedure, where lower gene expression levels were not detected. Thus, we retrospectively evaluated whether they were predictive markers for the response to S-1 and/or prognostic markers for patients enrolled in the ACTS-GC.

## Materials and methods

### Study population and design

Tumor tissue was collected from patients enrolled in the ACTS-GC, the inclusion criteria and treatment protocol of which have been described previously [4, 5]. After the completion of the first interim analysis of the ACTS-GC, this biomarker study was designed retrospectively to determine any predictive value for the benefit of S-1 treatment or for prognosis. The protocol used for the current biomarker study was approved by the ethics committee of the Japanese Gastric Cancer Association and the institutional review board of each participating hospital, and complied with the Reporting Recommendations for Tumor Marker Prognostic Studies (REMARK) guidelines [8].

### Reverse-transcription PCR

Hematoxylin and eosin-stained slides from formalin-fixed, paraffin-embedded (FFPE) specimens were reviewed by a pathologist to estimate the tumor load. Sections (10 μm thick) were then stained with nuclear fast red (Sigma-Aldrich, St. Louis, MO, USA) for manual microdissection. Tumor tissue was selected at a magnification of 5× to 10× and dissected using a scalpel, as described previously [9]. RNA was isolated from tumor tissue and cDNA was prepared as described previously [9], with a slight modification in the extraction step, which used RNeasy Mini Elute spin-columns (Qiagen, Chatsworth, GA, USA). The expression levels of 63 genes were determined using TaqMan real-time PCR (TaqMan array card; Life Technologies, Foster City, CA, USA) after TaqMan assay-based pre-amplification. Briefly, cDNA (2.5 µl) was pre-amplified using TaqMan PreAmp Master Mix (2×) (Life Technologies) and a pool of TaqMan Gene Expression Assays (0.2×) in a 10-µl polymerase chain reaction (PCR). The pre-amplification cycling conditions were as follows: 95 °C for 10 min, followed by 14 cycles of 95 °C for 15 s, and 60 °C for 4 min. An amplified cDNA sample was diluted 20 times in TE buffer. Amplified cDNA (25 µl) was added to 25 µl RNase-free water and 50 µl 2× TaqMan Gene Expression Master Mix (Life Technologies). The mixture was then transferred to a loading port for the TaqMan low-density array (LDA). The LDA was centrifuged twice, sealed, and PCR amplification was performed using the Applied Biosystems Prism 7900HT Sequence Detection System (Life Technologies) under the following thermal cycling conditions: 50 °C for 2 min and 94.5 °C for 10 min, followed by 40 cycles of 97 °C for 30 s and 59.7 °C for 1 min. The LDA included *ACTB*, *GAPDH*, and *RPLP0* as references based on their proven role as housekeeping genes [10, 11]. The assay IDs used in the LDA are shown in supplemental Table S1. The cycle threshold (*C*_t_) value, which is inversely proportional to the amount of cDNA, was calculated. The gene expression (relative mRNA) levels were expressed as the ratios (the differences between the *C*_t_ values) between the gene of interest and the geometric mean of the reference genes, which provided a baseline measurement for the amount of mRNA isolated from a specimen. The expression levels of each gene were categorized as low or high based on the 50th percentile (median). The Minimum Information for Publication of Quantitative Real-Time PCR Experiments (MIQE) guidelines checklist used is shown in supplemental Table S2.

### Data processing and statistical analysis

To determine the stability of the reference genes, the geNORM algorithm (MS-Excel add-on-macro program) was used, as described previously by Vandesompele et al. [12]. The program calculates the gene stability measure *M* by determining the average pairwise variation between a particular reference gene and all other control genes. Using genes with *M* values lower than 1.5, a normalization factor was calculated based on the geometric mean of the expression levels of the selected genes. To control the quality, target genes with data obtained from more than 60 % of the samples were employed, and the rest were excluded from further analysis.

The categorical data were analyzed using the chi-square test. Either the Wilcoxon or the Kruskal–Wallis test was used to assess correlations between groups. Survival curves were estimated using the Kaplan–Meier product-limit method, and the statistical significance of differences between survival curves was assessed using the log-rank test. Univariate and multivariate survival analyses were performed using a Cox proportional hazards model. Results were considered statistically significant at *P* < 0.05. All statistical analyses used the SAS software package version 9.1, the JMP software version 8.01 (SAS Institute, Cary, NC, USA) and MS-Excel (add-on-macro program; Microsoft, Redmond, WA, USA). The Benjamini and Hochberg false discovery rate (FDR)-controlling procedure was employed for multiple comparisons. Correlations of gene expression and prognosis were considered statistically significant at FDR *P* < 0.10.

### Validation of the prognostic capability of selected genes in an independent data set

Publicly available Illumina-DASL gene expression and clinical data (RFS data only; OS data were not available) of 432 samples from gastric cancer patients in Asia were downloaded via the Gene Expression Omnibus (GEO) database accession number GSE26253 [13]. Raw data of the GSE26253 data set were loaded onto GeneSpring GX version 12.6 (Agilent Technologies, Santa Clara, CA, USA). Gene expression data were normalized by two strategies: “per chip normalization” and “per gene normalization.” For “per chip normalization,” all expression data on a chip were normalized to the 75th percentile of all values on that chip. For “per gene normalization,” the data for a given gene were normalized to the median expression level of that gene across all samples. The detection *P* value was utilized for subsequent data quality control (QC) procedures. According to gene expression levels summarized from QC-passed probes (detection *P* < 0.05, >50 % of samples), 297 stage II or III gastric cancers from all 432 samples were categorized into two groups (i.e., “High” or “Low,” compared to the median) and were subjected to survival analysis as previously mentioned.

## Results

### Patient characteristics

Archived FFPE specimens obtained by surgical resection were available for 829 (78.3 %) of the 1059 patients who were enrolled in the ACTS-GC at 65 centers and constituted the biomarker study population. A summary of the patient demographic data and tumor characteristics was published elsewhere (Supplemental Table 3) [4, 5]. The median patient age was 62 years (range, 27–80 years). There was no significant difference between the population used in the current biomarker study and the total population of the ACTS-GC, as previously reported [2].

### Gene expression

The gene expression stability measures for the reference genes were calculated as 0.916, 0.931, and 0.923 for *GAPDH*, *ACTB*, and *RPLP0*, respectively. The *M* values were lower than 1.5 for all three genes, indicating that they could be utilized to normalize the target genes. Four genes (*CDKNA2*, *EGF*, *IGF2*, *SEMA3B*) were excluded from further analysis because their expression levels were below the detection limit in less than 60 % of the samples (51.5 %, 23.6 %, 44.5 %, and 5.5 %, respectively). Thus, 59 of the 63 genes subjected to LDA passed the quality control criteria. The median success rate for the 59 genes measured was 98.6 % (range, 61.2–100 %).

### Overall correlation of gene expression and OS or relapse-free survival

**Fig. 1:**
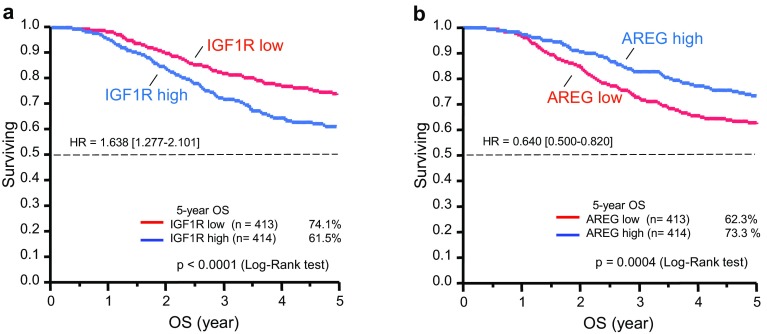
Kaplan–Meier curves showing overall survival (OS) for all patients according to *IGF1R* (**a**) and *AREG* (**b**) expression. OS was worse in tumors with high *IGF1R* and low *AREG*

Table 1 shows that among the 56 screened genes (excluding the three reference genes), *IGF1R* and *AREG* were most strongly correlated with OS (FDR, 0.0048 and 0.018, respectively). Kaplan–Meier plots of OS for all patients according to *IGF1R* and *AREG* expression levels are shown in Fig. 1. OS was significantly worse in high *IGF1R* patients than in low *IGF1R* patients, but better in high *AREG* patients than in low *AREG* patients. *IGF1R* was most strongly correlated with relapse-free survival (RFS; FDR, 0.007; Supplemental Table S4). Kaplan–Meier plots of the RFS of all patients according to *IGF1R* expression levels are shown in Supplemental Figure S1. RFS was significantly worse in high *IGF1R* patients than in low *IGF1R* patients.

**Table 1:** Univariate analysis of overall survival (OS) for all patients

Gene symbol	Log-rank *P*	BH-FDR_P	Hazard ratio	95 % low	95 % high
*IGF1R*	8.64E−05	0.005	1.64	1.28	2.10
*AREG*	3.70E−04	0.020	0.64	0.50	0.82
*ERBB2*	2.04E−03	0.110	1.47	1.15	1.88
*GZMA*	7.23E−03	0.383	0.70	0.54	0.91
*LRP5*	8.06E−03	0.419	1.40	1.09	1.79
*THBS1*	1.90E−02	0.968	1.34	1.05	1.71
*EZH2*	4.93E−02	0.979	0.78	0.60	1.00
*DAPK1*	5.37E−02	0.979	1.28	1.00	1.65
*UPP1*	5.78E−02	0.979	0.79	0.62	1.01
*CAV1*	5.80E−02	0.979	1.27	0.99	1.62
*ANGPT2*	6.17E−02	0.979	1.27	0.99	1.64
*DHFR*	7.83E−02	0.979	0.80	0.63	1.03
*TYMP*	8.34E−02	0.979	0.80	0.63	1.03
*DUT*	9.70E−02	0.979	0.81	0.64	1.04
*EREG*	9.70E−02	0.979	0.78	0.58	1.05
*SPARC*	1.25E−01	0.979	1.21	0.95	1.54
*MAPT*	1.33E−01	0.979	1.24	0.94	1.65
*EGFR*	1.42E−01	0.979	1.20	0.94	1.54
*FAS*	1.42E−01	0.979	0.83	0.64	1.07
*PTGS2*	1.58E−01	0.979	0.83	0.65	1.07
*PECAM1*	2.11E−01	0.979	1.17	0.92	1.49
*RRM1*	2.21E−01	0.979	1.17	0.91	1.49
*TGFA*	2.28E−01	0.979	1.17	0.91	1.51
*GADD45A*	2.36E−01	0.979	1.18	0.89	1.57
*MUC2*	2.39E−01	0.979	0.85	0.65	1.11
*HPSE*	2.71E−01	0.979	0.87	0.68	1.11
*TYMS*	2.91E−01	0.979	0.88	0.69	1.12
*RUNX3*	3.25E−01	0.979	0.88	0.69	1.13
*LDHA*	3.33E−01	0.979	0.89	0.69	1.13
*PTEN*	3.36E−01	0.979	0.89	0.69	1.13
*PLA2G2A*	3.70E−01	0.979	0.87	0.65	1.17
*REG4*	3.80E−01	0.979	0.89	0.70	1.15
*ABCC1*	4.00E−01	0.979	1.11	0.87	1.42
*TOP1*	4.07E−01	0.979	0.90	0.71	1.15
*ABCB1*	4.66E−01	0.979	0.91	0.70	1.18
*E2F1*	4.70E−01	0.979	0.91	0.71	1.17
*GGH*	5.91E−01	0.979	1.07	0.83	1.38
*FPGS*	6.15E−01	0.979	1.07	0.83	1.36
*TOP2A*	6.55E−01	0.979	1.06	0.83	1.35
*ITGB3*	6.70E−01	0.979	1.05	0.83	1.35
*BCL2L11*	6.75E−01	0.979	0.93	0.68	1.28
*APC*	6.95E−01	0.979	0.95	0.75	1.22
*ERCC1*	7.29E−01	0.979	1.04	0.82	1.33
*BCL2*	7.30E−01	0.979	1.05	0.80	1.38
*VCAM1*	7.40E−01	0.979	1.04	0.82	1.33
*RRM2*	7.71E−01	0.979	0.96	0.75	1.23
*MGMT*	7.79E−01	0.979	0.97	0.75	1.24
*BAX*	8.23E−01	0.979	0.97	0.76	1.24
*VEGFA*	8.34E−01	0.979	1.03	0.80	1.31
*DPYD*	8.47E−01	0.979	1.02	0.80	1.31
*UMPS*	8.55E−01	0.979	0.98	0.76	1.26
*ESR1*	9.12E−01	0.979	1.01	0.79	1.31
*MTHFR*	9.43E−01	0.979	1.01	0.78	1.31
*HDAC1*	9.68E−01	0.979	1.00	0.79	1.28
*PLAU*	9.70E−01	0.979	1.00	0.79	1.28
*MLH1*	9.79E−01	0.979	1.00	0.78	1.30

*BH-FDR* Benjamini & Hochberg false discovery rate

### Correlation of gene expression with OS or RFS in each treatment arm

Table 2 shows that among the 56 screened genes, only *IGF1R* was correlated with OS for patients who received surgery alone (FDR, 0.01). Kaplan–Meier plots of OS in the surgery-only arm are shown in Supplemental Figure S2A. OS was significantly worse in high *IGF1R* patients than in low *IGF1R* patients. No statistically significant correlations were detected between gene expression and OS in the S-1 arm. Supplemental Table S5 shows that *IGF1R* was correlated with RFS for patients who received surgery alone (FDR, 0.020). Kaplan–Meier plots of RFS in the surgery-alone arm are shown in Supplemental Figure S2B. RFS was significantly worse in high *IGF1R* patients than in low *IGF1R* patients. No statistically significant correlations were observed between gene expression and RFS in the S-1 arm.

**Table 2:** Univariate analysis of OS in surgery-only arm

Gene symbol	Log-rank *P*	BH-FDR_P	Hazard ratio	95 % low	95 % high
*IGF1R*	1.80E−04	0.010	1.848	1.333	2.562
*AREG*	2.20E−03	0.121	0.606	0.439	0.838
*LRP5*	1.42E−02	0.764	1.49	1.08	2.07
*ERBB2*	1.44E−02	0.764	1.49	1.08	2.05
*GZMA*	3.96E−02	0.982	0.70	0.50	0.98
*EZH2*	4.92E−02	0.982	0.72	0.52	1.00
*DHFR*	5.65E−02	0.982	0.73	0.53	1.01
*DAPK1*	6.01E−02	0.982	1.37	0.99	1.92
*TGFA*	6.04E−02	0.982	1.38	0.98	1.92
*EREG*	9.86E−02	0.982	0.72	0.48	1.07
*ANGPT2*	1.08E−01	0.982	1.31	0.94	1.84
*FPGS*	1.15E−01	0.982	1.29	0.94	1.79
*PLA2G2A*	1.24E−01	0.982	0.73	0.49	1.09
*ABCC1*	1.34E−01	0.982	1.28	0.93	1.77
*HPSE*	1.52E−01	0.982	0.79	0.57	1.09
*UPP1*	1.83E−01	0.982	0.80	0.58	1.11
*MTHFR*	2.07E−01	0.982	1.25	0.88	1.76
*MLH1*	2.16E−01	0.982	1.24	0.88	1.73
*LDHA*	2.33E−01	0.982	0.82	0.60	1.13
*GADD45A*	2.49E−01	0.982	1.24	0.86	1.78
*APC*	2.56E−01	0.982	1.20	0.87	1.65
*THBS1*	2.65E−01	0.982	1.20	0.87	1.65
*CAV1*	2.75E−01	0.982	1.19	0.87	1.64
*MAPT*	3.22E−01	0.982	1.21	0.83	1.76
*REG4*	3.31E−01	0.982	0.85	0.61	1.18
*PTGS2*	3.61E−01	0.982	0.86	0.62	1.19
*ABCB1*	3.83E−01	0.982	1.16	0.83	1.63
*EGFR*	3.94E−01	0.982	1.15	0.83	1.59
*SPARC*	4.04E−01	0.982	1.14	0.83	1.57
*RRM2*	4.10E−01	0.982	0.87	0.63	1.21
*BCL2*	4.10E−01	0.982	1.16	0.81	1.67
*MUC2*	4.16E−01	0.982	0.86	0.60	1.24
*DUT*	4.17E−01	0.982	0.88	0.64	1.21
*ERCC1*	4.34E−01	0.982	1.14	0.83	1.56
*PTEN*	5.04E−01	0.982	0.90	0.65	1.23
*HDAC1*	5.09E−01	0.982	0.90	0.65	1.24
*E2F1*	5.29E−01	0.982	1.11	0.80	1.54
*FAS*	5.60E−01	0.982	0.91	0.65	1.26
*RRM1*	5.61E−01	0.982	1.10	0.80	1.51
*TYMP*	6.08E−01	0.982	0.92	0.67	1.27
*VCAM1*	6.19E−01	0.982	0.92	0.67	1.27
*BAX*	6.91E−01	0.982	0.94	0.68	1.29
*ITGB3*	7.16E−01	0.982	1.06	0.77	1.46
*MGMT*	7.19E−01	0.982	1.06	0.77	1.47
*GGH*	7.35E−01	0.982	1.06	0.76	1.48
*PLAU*	7.47E−01	0.982	1.05	0.77	1.45
*TOP1*	8.07E−01	0.982	0.96	0.70	1.32
*UMPS*	8.28E−01	0.982	0.96	0.69	1.34
*RUNX3*	8.42E−01	0.982	1.03	0.75	1.42
*ESR1*	8.44E−01	0.982	0.97	0.69	1.35
*BCL2L11*	8.77E−01	0.982	0.97	0.64	1.45
*VEGFA*	8.90E−01	0.982	1.02	0.74	1.41
*TOP2A*	9.32E−01	0.982	0.99	0.72	1.36
*PECAM1*	9.46E−01	0.982	0.99	0.72	1.36
*DPYD*	9.79E−01	0.982	1.00	0.73	1.38
*TYMS*	9.82E−01	0.982	1.00	0.73	1.37

*BH-FDR* Benjamini & Hochberg false discovery rate

### Predictive value of biomarker analysis

**Fig. 2:**
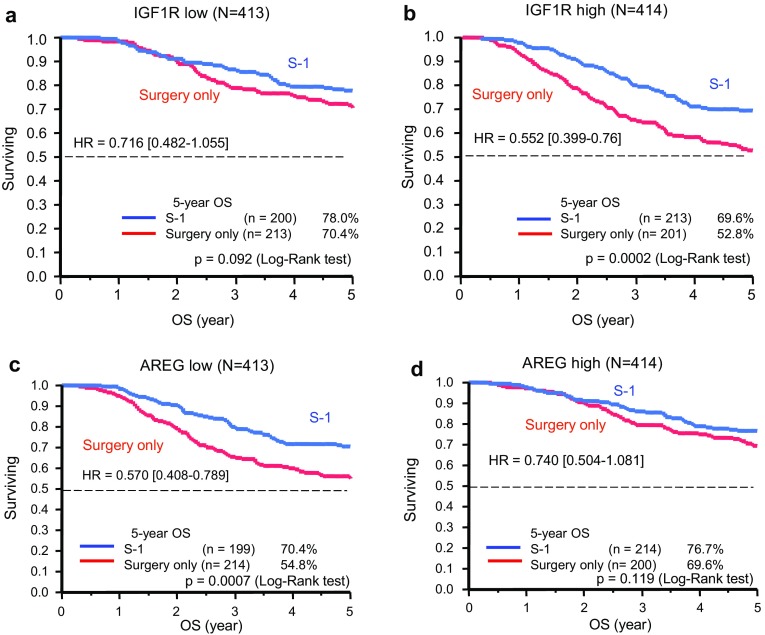
Kaplan–Meier curves showing OS for patients in the S-1-treated (*red*) and surgery-only (*blue*) groups for tumors with low *IGF1R* (**a**), high *IGF1R* (**b**), low *AREG* (**c**), and high *AREG* (**d**)

**Fig. 3:**
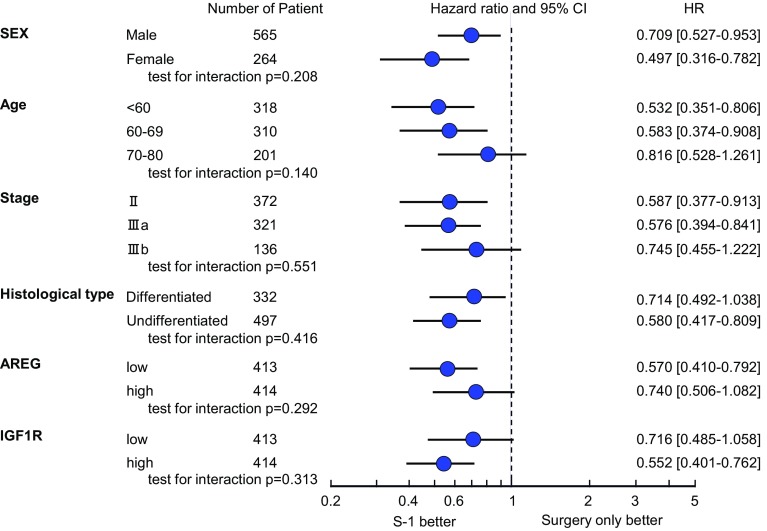
Hazard ratios (HRs) and 95 % confidence intervals (CIs) for OS in subgroups according to the levels of gene expression

Kaplan–Meier plots of OS for S-1 treatment versus surgery alone according to *IGF1R* and *AREG* expression levels are shown in Fig. 2a–d. The hazard ratio (HR) for death in the analysis of OS (S-1 vs. surgery alone) was lower in the high *IGF1R* group (HR, 0.55; 95 % CI, 0.40–0.76) than in the low *IGF1R* group (HR, 0.72; 95 % CI, 0.49–1.06). Similarly, the HR for death in the analysis of OS (S-1 vs. surgery alone) was much smaller in the low *AREG* group (HR, 0.57; 95 % CI, 0.41–0.79) than in the high *AREG* group (HR, 0.74; 95 % CI, 0.51–1.08). The prognostic relevance of *IGF1R* and *AREG* was assessed using a multivariate proportional hazards model adjusted for the following established clinical prognostic factors: treatment arm, gender, age, cancer stage, and histological type (Table 3). Although treatment arm and cancer stage were strong prognostic factors, *IGF1R* and *AREG* status were also independent prognostic factors. No statistically significant interactions were observed between *IGF1R* or *AREG* expression and S-1 treatment (Fig. 3).

**Table 3:** Multivariate analysis of OS

Group	Status	*N*	Hazard ratio (95 % CI)	*P* value
Arm	Surgery only	414	1	<0.0001
	S-1	412	0.593 (0.462–0.761)	
Sex	Female	263	1	0.740
	Male	563	0.955 (0.729–1.251)	
Age	<60 years	318	1	0.0017
	60–69 years	310	1.301 (1.104–1.532)	
	70–80 years	198	1.693 (1.219–2.347)	
Stage	II	372	1	<0.001
	IIIa	318	1.649 (1.402–1.940)	
	IIIb	136	2.719 (1.966–3.764)	
Histology	Differentiated	331	1	0.337
	Undifferentiated^a^	495	1.135 (0.876–1.471)	
AREG	Low	413	1	0.001
	High	413	0.658 (0.513–0.844)	
IGF1R	Low	412	1	<0.0001
	High	414	1.716 (1.332–2.212)	

^a^Including three patients with gastric cancer categorized as neither differentiated nor undifferentiated type

### Correlations among gene expressions and clinicopathological parameters

There was no statistically significant correlation between the mRNA expression levels of *IGF1R* and *AREG* (Spearman’s rank correlation coefficient: *r* = 0.035), and *AREG* and *EGFR* (*r* = 0.16). Any statistically significant relationship was not observed between clinicopathological parameters such as T, N, grade of differentiation, histological subtype, tumor size, and *IGF1R* and *AREG* gene expressions.

### Validation of prognostic capability of selected genes in an independent data set

QC-passed microarray data of an independent study cohort validated the prognostic capability of 44 genes of the 56 genes screened by LDA (Supplemental Table S6).

Twelve genes including *AREG* did not pass the QC procedure. Expression levels of *SPARC*, *EZH2*, *IGF1R*, and *E2F1* were strongly correlated with RFS (HR, 1.81, 0.63, 1.49, and 0.67; FDR, 0.06, 0.26, 0.34, and 0.34, respectively). Kaplan–Meier plots of RFS for all patients according to *SPARC*, *EZH2*, *IGF1R*, and *E2F1* expression levels are shown in Supplemental Figure S3. RFS was worse in patients with high *SPARC* or *IGF1R* than in patients with low *SPARC* or *IGF1R*, but better in patients with high *EZH2* or *E2F1* patients than in patients with low *EZH2* or *E2F1*.

## Discussion

This study retrospectively evaluated the influence of the expression levels of 63 preselected genes (including three reference genes) on the outcomes of patients enrolled in the ACTS-GC. We found an association between high *IGF1R* or low *AREG* expression and poor prognosis. We concluded that *IGF1R* and *AREG* are prognostic, not predictive, markers of stage II/III gastric cancer.

*IGF1R* is a multifunctional tyrosine kinase receptor that is activated by its ligands, *IGF1* and *IGF2*. *IGF1R* participates in several biological processes, including cell proliferation, differentiation, DNA repair, and prevention of apoptosis [14–17]. Aberrant activation of the IGF1/IGF1R axis has been associated with worse prognosis in many tumors, including breast, colorectal, laryngeal, myeloma, and prostate [18–20]. Data regarding *IGF1R* prognostic value in non-small cell lung cancer (NSCLC) are inconsistent [21–23]. Although relatively few gastric cancer cases have been evaluated, one report demonstrated that *IGFIR* overexpression in a primary tumor was correlated with increased lymph node metastasis, and that patients with low expression of both *IGF1R* and *EGFR* had significantly improved OS [24, 25]. In this study, *IGF1R* mRNA expression level was not correlated with tumor size, lymph node status, and staging of the tumors. *IGF2*, one of the ligands of *IGF1R*, could not be evaluated because its expression level was below the detection limit in less than 60 % of the samples. Previous papers that accounted for *IGF1R* analyzed relatively small numbers of samples in a retrospective manner, whereas the present study with its retrospective-prospective design enrolled 829 patients, showed the poor outcome of patients with high *IGF1R* expression, and successfully confirmed the prognostic value of this gene for gastric cancer. Furthermore, data from the publicly available database (GEO microarray data set) also supported the prognostic capability of *IGF1R* expression. Therefore, our results could encourage conducting further prospective studies to evaluate the IGF/IGFR axis.

AREG is a ligand for the epidermal growth factor receptor (EGFR), a transmembrane tyrosine kinase receptor that has a central role in regulating cell division and death [26]. AREG induces proliferative activities in various types of cells [27]. Recently, the effect of AREG on the prognosis and treatment efficacy of colorectal cancer patients receiving the anti-EGFR agent was investigated. High *AREG* or *EREG* expression identified a subgroup of *KRAS* wild-type patients who had a high probability of responding to EGFR inhibition [28]. The CO-17 study, which compared treatment with cetuximab and best supportive care (BSC) to BSC alone in patients with metastatic EGFR-positive colorectal cancer, revealed that *EREG* expression levels were positively correlated with cetuximab treatment efficacy [29]. Thus, AREG or EREG had a predictive value in patients treated with cetuximab. Interestingly, in patients with metastatic colorectal cancer receiving first-line chemotherapy without the anti-EGFR agent, high *AREG* or *EREG* expression significantly correlated with longer progression-free survival, and the positive prognostic value of high *EREG* was confirmed to be independent in a multivariate analysis [30]. Data regarding *AREG* prognostic values for NSCLC patients are inconsistent. Patients on the placebo arm with high AREG had statistically poorer OS than patients with low AREG, which remained significant in multivariate analysis, in the NCIC Clinical Trials Group BR.21 [31]. These discrepancies might depend on the difference of cancer type. We previously reported that patients with EGFR-positive tumors had worse survival than those with EGFR-negative tumors in the ACTS-GC biomarker study, when EGFR expression was evaluated by the immunohistochemical staining [4]. EGFR status had no relationship to *AREG* gene expression (data not shown). In addition, the prognostic values of *AREG* expression maintained in both patients with EGFR-positive (*n* = 75) and EGFR-negative (*n* = 752) tumors (data not shown). There have been few reports on *AREG* prognostic value in gastric cancer patients after surgery; the present ancestry study of the ACTS-GC is an important resource for evaluating the prognostic value of this gene.

The current study was limited by the following reasons. This study is for stage II and stage III patients, and this selection bias should be noticed to generalize our knowledge. The number of genes screened was relatively small. Additional useful candidate genes should be evaluated using archived cDNA from the present study in future investigations. Moreover, the correlation of gene expression according to mRNA measurement and protein levels should be further investigated using clinically feasible procedures such as immunohistochemical staining.

In conclusion, the current study provided compelling evidence that high *IGF1R* and low *AREG* expression were associated with poor prognosis after curative resection of stage II/III gastric cancer. There was no apparent interaction between S-1 and *IGF1R* or *AREG* status with respect to survival. These findings should contribute to the development of urgently required new targeted therapies for gastric cancer patients who are at high risk of relapse.

## Electronic supplementary material

Below is the link to the electronic supplementary material.

Supplementary material 1 (PDF 383 kb)

Supplementary material 2 (PDF 699 kb)
