# Supplementary material for: Impact of insulin-like growth factor-1 receptor and amphiregulin expression on survival in patients with stage II/III gastric cancer enrolled in the Adjuvant Chemotherapy Trial of S-1 for Gastric Cancer
Source: Gastric Cancer. 2016 Feb 16;20(2):263–73. doi: 10.1007/s10120-016-0600-x (PMC5321694; doi:10.1007/s10120-016-0600-x)
Supplement: Supplementary file 1 — Supplementary material 1 (PDF 383 kb) [file 10120_2016_600_MOESM1_ESM.pdf]

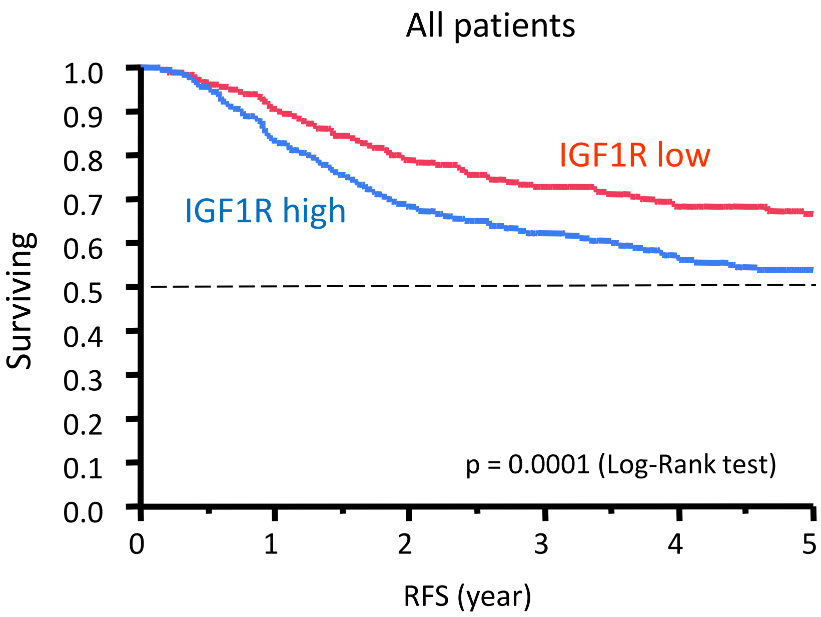

Supplemental Figure S1. Kaplan-Meier curves showing RFS for all patients according to *IGF1R* expression. RFS was poorer in high *IGF1R* tumors.

Gastric Cancer
Title: Impact of insulin-like growth factor 1 receptor and amphiregulin expression on survival in patients with stage II/III gastric cancer enrolled in the Adjuvant Chemotherapy Trial of S-1 for Gastric Cancer
Wataru Ichikawa, M.D., Ph.D., Division of Medical Oncology, Showa University Fujigaoka Hospital, 1-30 Fujigaoka, Aoba-ku, Yokohama, Kanagawa, 227-8501 Japan.
Tel.: +81-45-971-1151 Fax: +81-45-974-6319 E-mail: wataru@med.showa-u.ac.jp

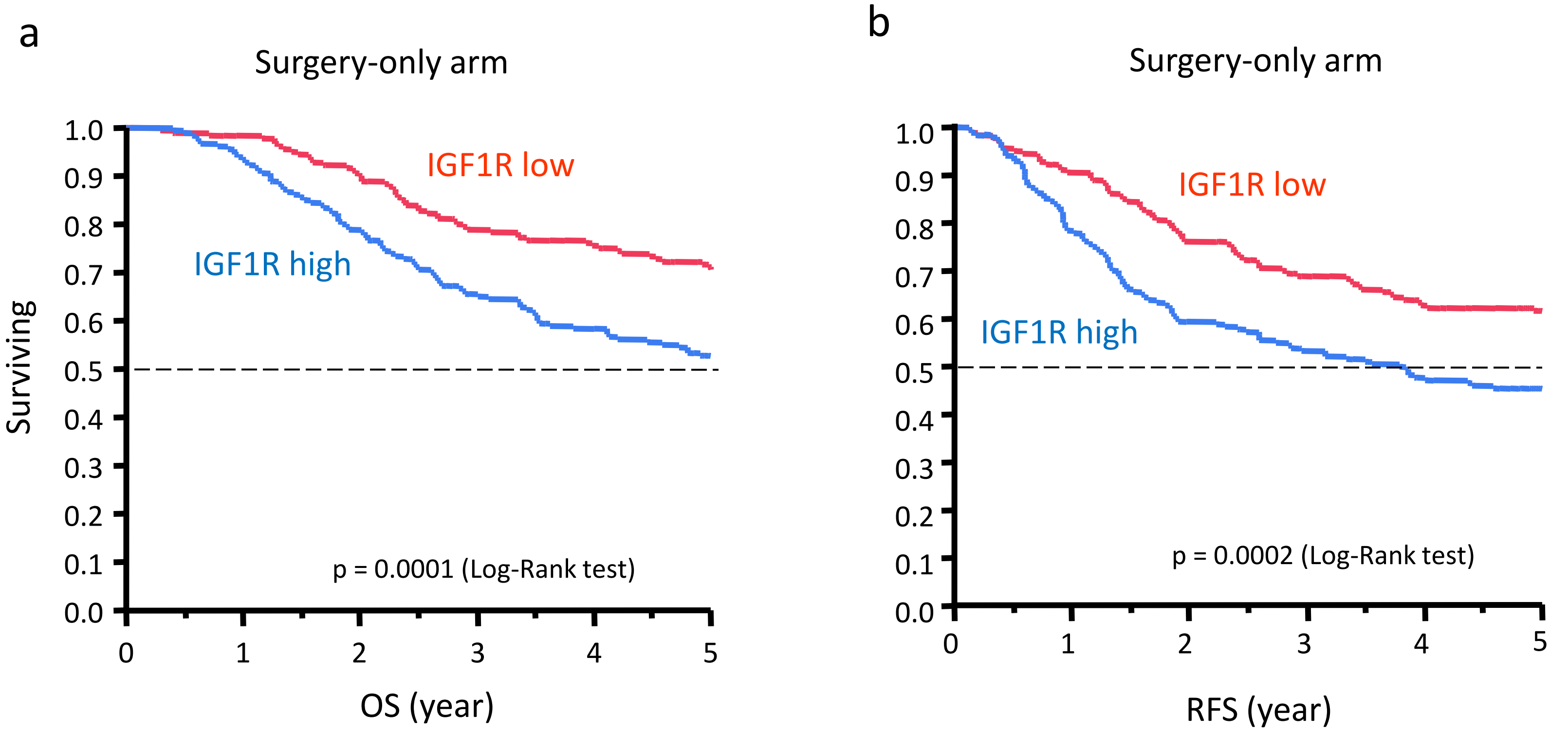

Supplemental Figure S2. Kaplan-Meier curves showing OS or RFS for patients in surgery-only arm according to *IGF1R* expression. OS (a) and RFS (b) in groups with tumors according to *IGF1R* expression. OS and RFS were poorer in high *IGF1R* tumors.

Gastric Cancer
Title: Impact of insulin-like growth factor 1 receptor and amphiregulin expression on survival in patients with stage II/III gastric cancer enrolled in the Adjuvant Chemotherapy Trial of S-1 for Gastric Cancer
Wataru Ichikawa, M.D., Ph.D., Division of Medical Oncology, Showa University Fujigaoka Hospital, 1-30 Fujigaoka, Aoba-ku, Yokohama, Kanagawa, 227-8501 Japan.
Tel.: +81-45-971-1151 Fax: +81-45-974-6319 E-mail: wataru@med.showa-u.ac.jp

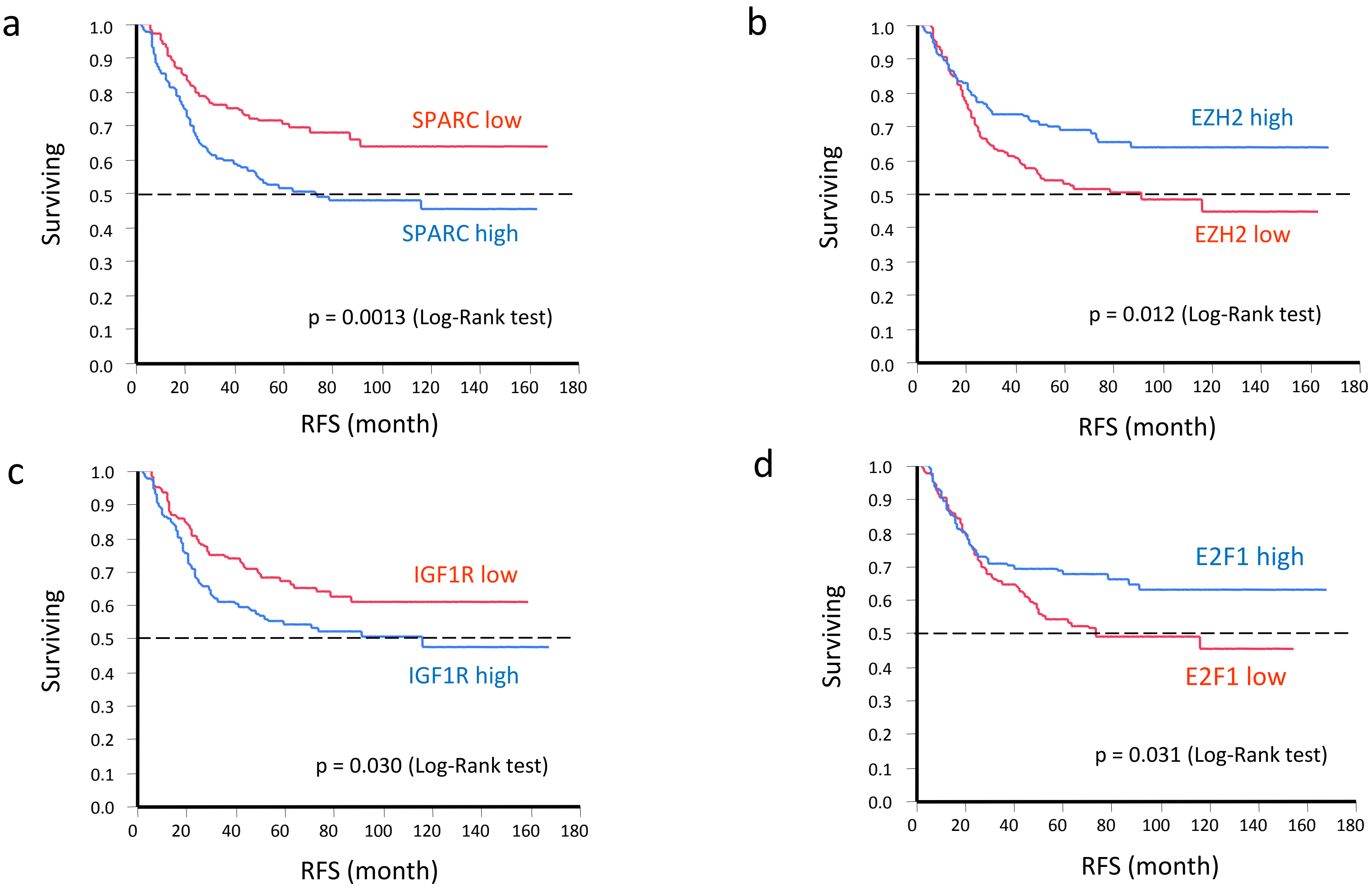

Supplemental Figure S3. Kaplan-Meier curves showing RFS for all patients according to *SPARC* (a), *EZH2* (b), *IGF1R* (c), and *E2F1* (d) expression from the GSE26253 data set. RFS was worse in tumors with high *SPARC* or *IGF1R* and low *EZH2* or *E2F1*.

Gastric Cancer
Title: Impact of insulin-like growth factor 1 receptor and amphiregulin expression on survival in patients with stage II/III gastric cancer enrolled in the Adjuvant Chemotherapy Trial of S-1 for Gastric Cancer
Wataru Ichikawa, M.D., Ph.D., Division of Medical Oncology, Showa University Fujigaoka Hospital, 1-30 Fujigaoka, Aoba-ku, Yokohama, Kanagawa, 227-8501 Japan.
Tel.: +81-45-971-1151 Fax: +81-45-974-6319 E-mail: wataru@med.showa-u.ac.jp
