# Supplementary material for: Impact of insulin-like growth factor-1 receptor and amphiregulin expression on survival in patients with stage II/III gastric cancer enrolled in the Adjuvant Chemotherapy Trial of S-1 for Gastric Cancer
Source: Gastric Cancer. 2016 Feb 16;20(2):263–73. doi: 10.1007/s10120-016-0600-x (PMC5321694; doi:10.1007/s10120-016-0600-x)
Supplement: Supplementary file 2 — Supplementary material 2 (PDF 699 kb) [file 10120_2016_600_MOESM2_ESM.pdf]

Table S1 Assay IDs of Low Density Array

Assay ID	Gene Symbol	Gene Name	Amplicon size (bp)
Hs03929097_g1	*GAPDH*	glyceraldehyde-3-phosphate dehydrogenase	58
Hs00184500_m1	*ABCB1*	ATP-binding cassette; sub-family B (MDR/TAP); member 1	67
Hs00219905_m1	*ABCC1*	ATP-binding cassette; sub-family C (CFTR/MRP); member 1	74
Hs00357333_g1	*ACTB*	actin; beta;	77
Hs00169867_m1	*ANGPT2*	angiopoietin 2	73
Hs00181051_m1	*APC*	adenomatous polyposis coli	62
Hs00950669_m1	*AREG*	amphiregulin	66
Hs00180269_m1	*BAX*	BCL2-associated X protein	62
Hs99999018_m1	*BCL2*	B-cell CLL/lymphoma 2	76
Hs00375807_m1	*BCL2L11*	BCL2-like 11 (apoptosis facilitator)	64
Hs00971716_m1	*CAV1*	caveolae protein; 22kDa	66
Hs99999189_m1	*CDKN2A*	cyclin-dependent kinase inhibitor 2A	72
Hs00234480_m1	*DAPK1*	death-associated protein kinase 1	76
Hs00758822_s1	*DHFR*	dihydrofolate reductase	75
Hs00559278_m1	*DPYD*	dihydropyrimidine dehydrogenase	71
Hs00984632_gH	*DUT*	deoxyuridine triphosphatase	87
Hs00153451_m1	*E2F1*	E2F transcription factor 1	84
Hs01099999_m1	*EGF*	epidermal growth factor	70
Hs01076078_m1	*EGFR*	epidermal growth factor receptor	60
Hs01001580_m1	*ERBB2*	v-erb-b2 erythroblastic leukemia viral oncogene homolog 2	60
Hs01012158_m1	*ERCC1*	excision repair cross-complementing rodent repair deficiency	55
Hs00914313_m1	*EREG*	epiregulin	65
Hs00174860_m1	*ESR1*	estrogen receptor 1	62
Hs00544830_m1	*EZH2*	enhancer of zeste homolog 2 (Drosophila)	86
Hs00163653_m1	*FAS*	Fas (TNF receptor superfamily; member 6)	85
Hs00909424_g1	*FPGS*	folylpolyglutamate synthase	85
Hs00169255_m1	*GADD45A*	growth arrest and DNA-damage-inducible; alpha;	123
Hs00914163_m1	*GGH*	gamma-glutamyl hydrolase	91
Hs00989184_m1	*GZMA*	granzyme A	56
Hs02621185_s1	*HDAC1*	histone deacetylase 1	103
Hs00180737_m1	*HPSE*	heparanase	59
Hs00609566_m1	*IGF1R*	insulin-like growth factor 1 receptor	64

Assay ID	Gene Symbol	Gene Name	Amplicon size (bp)
Hs00171254_m1	*IGF2*	insulin-like growth factor 2	93
Hs01001469_m1	*ITGB3*	beta 3 (platelet glycoprotein IIIa; antigen CD61)	59
Hs00855332_g1	*LDHA*	lactate dehydrogenase A	130
Hs00182031_m1	*LRP5*	low density lipoprotein receptor-related protein 5	73
Hs00902194_m1	*MAPT*	microtubule-associated protein tau	59
Hs01037698_m1	*MGMT*	O-6-methylguanine-DNA methyltransferase	71
Hs00179866_m1	*MLH1*	mutL homolog 1; colon cancer; nonpolyposis type 2 (E. coli)	103
Hs01114487_m1	*MTHFR*	methylenetetrahydrofolate reductase (NAD(P)H)	87
Hs03005094_m1	*MUC2*	mucin 2; oligomeric mucus/gel-forming	64
Hs00169777_m1	*PECAM1*	platelet/endothelial cell adhesion molecule 1	65
Hs01047228_m1	*PLA2G2A*	phospholipase A2; group IIA (platelets; synovial fluid)	84
Hs01547054_m1	*PLAU*	plasminogen activator; urokinase	61
Hs02621230_s1	*PTEN*	phosphatase and tensin homolog	135
Hs00153133_m1	*PTGS2*	prostaglandin-endoperoxide synthase 2 (prostaglandin G/H synthase and cyclooxygenase)	75
Hs00230746_m1	*REG4*	regenerating islet-derived family; member 4	58
Hs00420895_gH	*RPLP0*	ribosomal protein; large; P0	76
Hs00168784_m1	*RRM1*	ribonucleotide reductase M1	78
Hs00357247_g1	*RRM2*	ribonucleotide reductase M2	79
Hs00231709_m1	*RUNX3*	runt-related transcription factor 3	77
Hs00190328_m1	*SEMA3B*	immunoglobulin domain (Ig); short basic domain; secreted; (semaphorin) 3B	67
Hs00234160_m1	*SPARC*	secreted protein; acidic; cysteine-rich (osteonectin)	76
Hs00608187_m1	*TGFA*	transforming growth factor; alpha	70
Hs00962908_m1	*THBS1*	thrombospondin 1	59
Hs00243257_m1	*TOP1*	topoisomerase (DNA) I	101
Hs03063307_m1	*TOP2A*	topoisomerase (DNA) II alpha 170kDa	72
Hs00157317_m1	*TYMP*	thymidine phosphorylase	95
Hs00426586_m1	*TYMS*	thymidylate synthetase	60
Hs00923517_m1	*UMPS*	uridine monophosphate synthetase	85
Hs01066247_m1	*UPP1*	uridine phosphorylase 1	68
Hs01003372_m1	*VCAM1*	vascular cell adhesion molecule 1	62
Hs00900055_m1	*VEGFA*	vascular endothelial growth factor A	59

Gastric Cancer
Title: Impact of insulin-like growth factor 1 receptor and amphiregulin expression on survival in patients with stage II/III gastric cancer enrolled in the Adjuvant Chemotherapy Trial of S-1 for Gastric Cancer
Wataru Ichikawa, M.D., Ph.D., Division of Medical Oncology, Showa University Fujigaoka Hospital, 1-30 Fujigaoka, Aoba-ku, Yokohama, Kanagawa, 227-8501 Japan.
Tel.: +81-45-971-1151 Fax: +81-45-974-6319 E-mail: wataru@med.showa-u.ac.jp

Table S2 MIQE guidelines check list

ITEM TO CHECK	IMPORTANCE	CHECKLIST	Memo
**EXPERIMENTAL DESIGN**			
Definition of experimental and control groups	**E**	**Checked**	Surgery only arm vs. S-1 treatment arm
Number within each group	**E**	**Checked**	Surgery only (414) vs. S-1 treatment (412)
Assay carried out by core lab or investigator's lab?	D	Checked	Falco biosystems Co., Ltd. Kyoto-City, Japan
Acknowledgement of authors' contributions	D	Checked	
**SAMPLE**			
Description	**E**	Checked	
Volume/mass of sample processed	D	Checked	FFPE specimens (10um thick / up to 5 slices)
Microdissection or macrodissection	**E**	Checked	Macrodissected samples
Processing procedure	**E**	Checked	
If frozen - how and how quickly?	**E**	Checked	
If fixed - with what, how quickly?	**E**	Checked	
Sample storage conditions and duration (especially for FFPE samples)	**E**	Checked	various period of time of storage (65 institutions/ archived block)
**NUCLEIC ACID EXTRACTION**			
Procedure and/or instrumentation	**E**	Checked	
Name of kit and details of any modifications	**E**	Checked	Rnaeasy mini kit (QIAGEN)
Source of additional reagents used	D	Checked	none
Details of DNase or RNAse treatment	**E**	Checked	Dnase I digestion on spin column
Contamination assessment (DNA or RNA)	**E**	Checked	Gene Specific probe / primer design
Nucleic acid quantification	**E**	Checked	
Instrument and method	**E**	Checked	Nano-drop system
Purity (A260/A280)	D	NA	NA
Yield	D	NA	NA
RNA integrity method/instrument	**E**	NA	NA
RIN/RQI or Cq of 3' and 5' transcripts	**E**	NA	NA
Electrophoresis traces	D	NA	NA
Inhibition testing (Cq dilutions, spike or other)	**E**		
**REVERSE TRANSCRIPTION**			
Complete reaction conditions	**E**	Checked	According to the manifacture's protocol
Amount of RNA and reaction volume	**E**	Checked	2.5ug total RNA
Priming oligonucleotide (if using GSP) and concentration	**E**	Checked	Random hexamer (High-Capacity cDNA Prverse Transcription kits(Applied Biosystems)
Reverse transcriptase and concentration	**E**	Checked	According to kit protocol
Temperature and time	**E**	Checked	25degree 10min, 37 degree 2hours, 85degree 5 sec
Manufacturer of reagents and catalogue numbers	D	Checked	ABI Catalogue number = 4368814 / 200 reaction
Cqs with and without RT	D*		
Storage conditions of cDNA	D	Checked	below - 20 degree celsius
**qPCR TARGET INFORMATION**			
If multiplex, efficiency and LOD of each assay.	**E**	NA	NA
Sequence accession number	**E**	Checked	Despcibed in supplemental table
Location of amplicon	D		
Amplicon length	**E**	Checked	Despcibed in supplemental table
In silico specificity screen (BLAST, etc)	**E**	Checked	ABI designed taqman assay (Low Density Card) were employed.
Pseudogenes, retropseudogenes or other homologs?	D		92.1% of assay are "m1", namely mRNA specific, whose probe spans an exon-exon junction and will not detect genomic DNA.
Sequence alignment	D		3.6% of assay are "g1", whose probe spans an exon-exon junction, however, the assay may detect genomic DNA if present in the sample.
Secondary structure analysis of amplicon	D		4.2% of assay are "s1", whose primers and probes are designed within a single exon, such assays will, by devinition detect genomic DNA.
Location of each primer by exon or intron (if applicable)	**E**	Checked	Exon
What splice variants are targeted?	**E**	Checked	As described above
**qPCR OLIGONUCLEOTIDES**			
Primer sequences	**E**	Checked	Sequence information is not disclosed by ABI
RTPrimerDB Identification Number	D		Sequence information is not disclosed by ABI
Probe sequences	D**		Sequence information is not disclosed by ABI
Location and identity of any modifications	**E**	Checked	Sequence information is not disclosed by ABI
Manufacturer of oligonucleotides	D	Checked	
Purification method	D		
**qPCR PROTOCOL**			
Complete reaction conditions	**E**	Checked	According to the manifacture's protocol
Reaction volume and amount of cDNA/DNA	**E**	Checked	1ng cDNA / 1ul of reaction mixture, Reaction volume per well = 3ul
Primer, (probe), Mg++ and dNTP concentrations	**E**	Checked	Not disclosed by ABI
Polymerase identity and concentration	**E**	Checked	Primer (900nM), Probe (250nM)
Buffer/kit identity and manufacturer	**E**	Checked	TaqMan Gene Expression Master Mix (Parts Number : 4369016)
Exact chemical constitution of the buffer	D	Checked	Not disclosed by ABI
Additives (SYBR Green I, DMSO, etc.)	**E**	Checked	
Manufacturer of plates/tubes and catalog number	D	Checked	TaqMan Arrays (384-well micro Fluidic cards Parts Number = MFC_384)
Complete thermocycling parameters	**E**	Checked	2 min at 50°C and 10 min at 94.5°C for 40 cycles (30 s at 97°C and 1 min at 59.7°C).
Reaction setup (manual/robotic)	D	Checked	Manual
Manufacturer of qPCR instrument	**E**	Checked	7900HT Fast Preal-Time PCR System
**qPCR VALIDATION**			
Evidence of optimisation (from gradients)	D		
Specificity (gel, sequence, melt, or digest)	**E**	Checked	This issue may not be suitable for 384-well micro Fluidic cards system. However Applied Biosystems reccomend double-delta Ct method for this system, namely, standard curve and PCR efficacy are well controled in this sytems.
For SYBR Green I, Cq of the NTC	**E**	Checked	NTC well assigned
Standard curves with slope and y-intercept	**E**	Checked	This issue may not be suitable for 384-well micro Fluidic cards system. However Applied Biosystems reccomend double-delta Ct method for this system, namely, slope of standard curve are nealy -3.32 for all primers.
PCR efficiency calculated from slope	**E**	Checked	This issue may not be suitable for 384-well micro Fluidic cards system. However Applied Biosystems reccomend double-delta Ct method for this system, therefore, efficacy of reaction are nealy 100% for all primers.
Confidence interval for PCR efficiency or standard error	D		
r2 of standard curve	**E**	Checked	Double delta Ct method employed (ABI confirmed)
Linear dynamic range	**E**	Checked	Double delta Ct method employed (ABI confirmed)
Cq variation at lower limit	**E**	Checked	Double delta Ct method employed (ABI confirmed)
Confidence intervals throughout range	D		
Evidence for limit of detection	**E**	Checked	Double delta Ct method employed (ABI confirmed)
If multiplex, efficiency and LOD of each assay.	**E**	Checked	Not multiplex assay
**DATA ANALYSIS**			
qPCR analysis program (source, version)	**E**	Checked	Sequence ditection software (ABI)
Cq method determination	**E**	Checked	Auto threshould
Outlier identification and disposition	**E**	Checked	Outlier rejection emabled on the software.
Results of NTCs	**E**	Checked	No amplification in NTC confirmed
Justification of number and choice of reference genes	**E**	Checked	Eight house-keeping gene were utilized. (ACTB, B2M, GAPDH , GUSB, HMBS, RPLP0, YWHAZ and 18S)
Description of normalisation method	**E**	Checked	Geometric mean of eight house-keeping genes were employed followed by log 2 transformation.
Number and concordance of biological replicates	D	Checked	Ct deferences of duplicated data were checked.
Number and stage (RT or qPCR) of technical replicates	**E**	Checked	Ct deferences of duplicated data were checked.
Repeatability (intra-assay variation)	E	Checked	Ct deferences of duplicated data were checked.
Reproducibility (inter-assay variation, %CV)	D		
Power analysis	D	NA	Exploratory study design (not priory power calculation)
Statistical methods for result significance	**E**	Checked	Cox proportional hazard model, Log-Rank test, (Benjamini and Hochberg false discovery rate (FDR) controlling procedure)
Software (source, version)	E	Checked	SAS ver 8 & JMP software (SAS institute, JAPAN)
Cq or raw data submission using RDML	**D**		

Table 1. MIQE checklist for authors, reviewers and editors. All essential information (E) must be submitted with the manuscript. Desirable

information (D) should be submitted if available. If using primers obtained from RTPrimerDB, information on qPCR target, oligonucleotides, protocols and validation is available from that source.

*: Assessing the absence of DNA using a no RT assay is essential when first extracting RNA. Once the sample has been validated as RDNA-free, inclusion of a no-RT control is desirable, but no longer essential.

Table S3

Characteristics of the patients

	S-1 (*n* = 415)	Surgery only (*n* = 414)	P value [a]
Sex, *n* (%)			0.9
Male	282 (68.0)	283 (68.4)	
Female	133 (32.0)	131 (31.6)	
Age, *n* (%)			0.72
<60	160 (38.6)	158 (38.2)	
60–69	149 (35.9)	161 (38.9)	
70–80	106 (25.5)	95 (22.9)	
Median, y	63	62	
Range, y	27–80	33–80	
Tumor stage, *n* (%)			0.93
T1	1 (0)	0 (0)	
T2	222 (53.5)	223 (53.9)	
T3	180 (43.4)	182 (44.0)	
T4	12 (2.9)	9 (2.2)	
Nodal stage, n (%) [b]			0.52
N0	40 (9.6)	52 (12.6)	
N1	233 (56.1)	222 (53.6)	
N2	142 (34.2)	140 (33.8)	
N3	0 (0)	0 (0)	
Lymph-node metastases, *n* (%)		0.18	
0	40 (9.6)	52 (12.6)	
1–6	254 (61.2)	254 (61.4)	
7–15	97 (23.4)	85 (20.5)	
≥16	24 (5.8)	23 (5.6)	
Cancer stage, n (%) [c]			0.48
II	183 (44.1)	189 (45.7)	
IIIA	159 (38.3)	162 (39.1)	
IIIB	73 (17.6)	63 (15.2)	
IV	0 (0)	0 (0)	
Histologic type, n (%) [d]			0.91
Differentiated	166 (40.0)	166 (40.1)	
Undifferentiated	249 (60.0)	245 (59.2)	

a) P values for sex were calculated with the use of the χ2 test. P values for age, tumor stage, nodal stage, number of lymph-node metastases, cancer stage (Japanese classification), and histologic type were calculated with the use of the Wilcoxon test.

b) Nodal stages according to the Japanese classification were defined as follows: N0, no evidence of lymph node metastasis; N1, metastasis to group 1 lymph nodes; N2, metastasis to group 2 lymph nodes; N3, metastasis to group 3 lymph nodes. Groups 1, 2, and 3 are regional lymph node classifications defined according to the location of the primary tumor and are based on the results of studies of lymphatic flow at various tumor sites and the observed survival associated with metastasis at each nodal station (i.e., position in relation to primary node).

c) Cancer stages according to the Japanese classification were defined as follows: stage IA, T1N0; stage IB, T1N1 or T2N0; stage II, T1N2, T2N1, or T3N0; stage IIIA, T2N2, T3N1, or T4N0; stage IIIB, T3N2 or T4N1; stage IV, T4N2, any T stage with N3, or distant metastasis.

d) In entire population of ACTS-GC, histologic type was classified among eligible patients (n = 1,034). In the surgery-only group of biomarker study population, cancers could not be classified as differentiated or undifferentiated in 3 patients.

Table S4 Univariate analysis of RFS for all patients

Gene Symbol	Log-Rank P	BH-FDR_P	Hazard ratio	95%Low	95%High
IGF1R	1.30E-04	0.007	1.539	1.232	1.922
THBS1	2.13E-03	0.117	1.413	1.132	1.763
ERBB2	2.71E-03	0.146	1.40	1.12	1.74
AREG	5.88E-03	0.312	0.73	0.59	0.92
LRP5	8.33E-03	0.433	1.35	1.08	1.68
EGFR	1.01E-02	0.517	1.34	1.07	1.68
GZMA	1.80E-02	0.900	0.76	0.60	0.95
EZH2	4.98E-02	0.920	0.80	0.63	1.00
MAPT	5.33E-02	0.920	1.28	1.00	1.65
PTEN	5.45E-02	0.920	0.81	0.65	1.00
FAS	5.87E-02	0.920	0.80	0.64	1.01
ANGPT2	6.16E-02	0.920	1.24	0.99	1.56
TGFA	7.20E-02	0.920	1.23	0.98	1.55
MUC2	8.36E-02	0.920	0.81	0.63	1.03
CAV1	8.55E-02	0.920	1.21	0.97	1.51
PECAM1	9.57E-02	0.920	1.20	0.97	1.50
LDHA	1.02E-01	0.920	0.83	0.67	1.04
TYMP	1.05E-01	0.920	0.83	0.67	1.04
SPARC	1.07E-01	0.920	1.20	0.96	1.49
UMPS	1.23E-01	0.920	0.84	0.67	1.05
UPP1	1.28E-01	0.920	0.84	0.67	1.05
ABCC1	1.32E-01	0.920	1.19	0.95	1.48
ERCC1	1.35E-01	0.920	1.18	0.95	1.47
RUNX3	1.63E-01	0.920	0.85	0.68	1.07
GADD45A	2.05E-01	0.920	1.18	0.91	1.52
DHFR	2.06E-01	0.920	0.87	0.70	1.08
DAPK1	2.07E-01	0.920	1.16	0.92	1.45
TOP1	2.15E-01	0.920	0.87	0.70	1.08

Gene Symbol	Log-Rank P	BH-FDR_P	Hazard ratio	95%Low	95%High
DUT	2.29E-01	0.920	0.87	0.70	1.09
TYMS	2.41E-01	0.920	0.88	0.70	1.09
PLA2G2A	2.43E-01	0.920	0.85	0.66	1.11
PTGS2	3.48E-01	0.920	0.90	0.72	1.12
EREG	3.54E-01	0.920	0.88	0.68	1.15
HPSE	4.42E-01	0.920	0.92	0.73	1.14
GGH	4.45E-01	0.920	1.09	0.87	1.38
FPGS	4.54E-01	0.920	1.09	0.87	1.36
ABCB1	4.81E-01	0.920	0.92	0.73	1.16
MLH1	4.98E-01	0.920	0.92	0.73	1.16
TOP2A	5.32E-01	0.920	1.07	0.86	1.34
ITGB3	5.52E-01	0.920	1.07	0.86	1.33
ESR1	5.79E-01	0.920	0.94	0.75	1.18
VEGFA	5.88E-01	0.920	1.06	0.85	1.32
RRM1	6.30E-01	0.920	1.06	0.85	1.32
REG4	6.45E-01	0.920	0.95	0.76	1.19
HDAC1	6.68E-01	0.920	0.95	0.76	1.19
BCL2	6.78E-01	0.920	1.05	0.83	1.34
DPYD	6.87E-01	0.920	1.05	0.84	1.31
BCL2L11	6.98E-01	0.920	0.95	0.71	1.25
RRM2	7.45E-01	0.920	0.96	0.77	1.20
APC	8.29E-01	0.920	1.02	0.82	1.28
PLAU	8.31E-01	0.920	0.98	0.78	1.22
E2F1	8.39E-01	0.920	0.98	0.78	1.23
MGMT	8.96E-01	0.920	0.99	0.79	1.23
BAX	9.02E-01	0.920	0.99	0.79	1.23
MTHFR	9.17E-01	0.920	1.01	0.80	1.28
VCAM1	9.20E-01	0.920	1.01	0.81	1.26

BH-FDR_P : Benjamini & Hochberg false discovery rate

Table S5 Univariate analysis of RFS for surgery-only arm

Gene Symbol	Log-Rank P	BH-FDR_P	Hazard ratio	95%Low	95%High
IGF1R	3.58E-04	0.020	1.692	1.263	2.266
ERBB2	8.08E-03	0.444	1.475	1.104	1.970
EZH2	1.24E-02	0.669	0.68	0.51	0.92
ERCC1	1.50E-02	0.795	1.43	1.07	1.91
LRP5	2.19E-02	0.980	1.40	1.05	1.87
THBS1	3.28E-02	0.980	1.37	1.02	1.83
LDHA	3.38E-02	0.980	0.73	0.55	0.98
AREG	5.06E-02	0.980	0.75	0.56	1.00
ANGPT2	6.18E-02	0.980	1.33	0.99	1.80
TGFA	7.11E-02	0.980	1.32	0.98	1.78
EGFR	8.11E-02	0.980	1.30	0.97	1.73
PTEN	8.27E-02	0.980	0.78	0.58	1.03
APC	9.41E-02	0.980	1.28	0.96	1.71
HPSE	1.07E-01	0.980	0.79	0.59	1.05
MAPT	1.22E-01	0.980	1.30	0.93	1.82
PLA2G2A	1.42E-01	0.980	0.77	0.54	1.09
ABCC1	1.43E-01	0.980	1.24	0.93	1.67
MUC2	1.70E-01	0.980	0.80	0.57	1.10
EREG	1.82E-01	0.980	0.79	0.56	1.12
E2F1	1.83E-01	0.980	1.22	0.91	1.65
DHFR	1.87E-01	0.980	0.82	0.62	1.10
UPP1	1.95E-01	0.980	0.83	0.62	1.10
SPARC	2.11E-01	0.980	1.20	0.90	1.60
FAS	2.14E-01	0.980	0.83	0.61	1.12
TOP1	2.24E-01	0.980	0.84	0.63	1.12
GZMA	2.37E-01	0.980	0.83	0.61	1.13
UMPS	2.39E-01	0.980	0.84	0.62	1.13
TYMP	2.54E-01	0.980	0.84	0.63	1.13

Gene Symbol	Log-Rank P	BH-FDR_P	Hazard ratio	95%Low	95%High
MTHFR	3.08E-01	0.980	1.18	0.86	1.61
FPGS	3.15E-01	0.980	1.16	0.87	1.55
RRM2	3.43E-01	0.980	0.87	0.65	1.16
BCL2	3.52E-01	0.980	1.17	0.84	1.61
CAV1	3.62E-01	0.980	1.14	0.86	1.52
PTGS2	3.65E-01	0.980	0.87	0.65	1.17
HDAC1	3.69E-01	0.980	0.88	0.66	1.17
DAPK1	3.82E-01	0.980	1.14	0.85	1.54
DUT	3.92E-01	0.980	0.88	0.66	1.18
ABCB1	3.97E-01	0.980	1.14	0.84	1.55
REG4	4.07E-01	0.980	0.88	0.66	1.19
VEGFA	4.10E-01	0.980	1.13	0.85	1.50
GGH	4.35E-01	0.980	1.13	0.83	1.53
ESR1	4.45E-01	0.980	0.89	0.66	1.20
GADD45A	5.98E-01	0.980	1.09	0.79	1.52
PECAM1	6.23E-01	0.980	1.07	0.81	1.43
ITGB3	6.52E-01	0.980	1.07	0.80	1.42
MGMT	7.03E-01	0.980	1.06	0.79	1.42
DPYD	7.13E-01	0.980	0.95	0.71	1.26
TYMS	7.23E-01	0.980	0.95	0.71	1.27
PLAU	7.79E-01	0.980	0.96	0.72	1.28
RRM1	8.11E-01	0.980	1.04	0.78	1.38
TOP2A	8.72E-01	0.980	1.02	0.77	1.37
BCL2L11	9.43E-01	0.980	0.99	0.68	1.42
VCAM1	9.57E-01	0.980	1.01	0.76	1.34
RUNX3	9.57E-01	0.980	0.99	0.74	1.33
MLH1	9.70E-01	0.980	1.01	0.75	1.36
BAX	9.80E-01	0.980	1.00	0.75	1.34

BH-FDR_P : Benjamini & Hochberg false discovery rate

Table S6 Univariate analysis of RFS (44 genes screened by LDA on publicly available dataset; GSE26253)

Gene	Hazard Ratio	HR: 95%CI-low	HR: 95%CI-high	Log-Rank P	FDR P	3-yr RFS (Low-group)	3-yr RFS (High-group)
SPARC	1.816	1.255	2.629	0.001	0.058	76.3	59.9
EZH2	0.628	0.436	0.906	0.012	0.262	62.2	73.8
IGF1R	1.489	1.037	2.139	0.030	0.336	74.6	61.3
E2F1	0.671	0.466	0.966	0.031	0.336	65.0	71.1
RUNX3	0.711	0.495	1.020	0.063	0.414	65.9	70.0
DHFR	1.397	0.974	2.004	0.067	0.414	72.8	63.0
RRM2	0.720	0.502	1.035	0.074	0.414	62.6	73.6
PTGS2	1.388	0.965	1.995	0.075	0.414	71.0	65.1
DUT	1.353	0.943	1.942	0.099	0.485	71.5	64.5
ABCC1	0.751	0.524	1.078	0.119	0.525	65.4	70.6
MTHFR	1.311	0.915	1.880	0.138	0.542	70.3	65.5
VCAM1	1.293	0.901	1.853	0.162	0.542	70.9	65.2
REG4	0.779	0.544	1.117	0.173	0.542	64.6	71.4
FAS	1.272	0.887	1.824	0.190	0.542	72.4	63.7
VEGFA	1.253	0.875	1.794	0.217	0.542	73.1	62.8
GZMA	0.799	0.557	1.145	0.220	0.542	64.6	71.6
ANGPT2	1.250	0.873	1.790	0.222	0.542	71.5	64.5
FPGS	0.800	0.558	1.146	0.222	0.542	63.3	72.8
TOP2A	0.813	0.567	1.165	0.258	0.598	64.8	71.4
PLA2G2A	0.825	0.576	1.181	0.292	0.618	67.3	68.6
ERCC1	1.209	0.844	1.731	0.299	0.618	71.6	64.5
PLAU	1.204	0.841	1.722	0.309	0.618	71.8	64.1
LRP5	0.835	0.583	1.197	0.325	0.622	66.6	69.5
DAPK1	1.168	0.816	1.672	0.396	0.726	70.5	65.5
UPP1	0.871	0.608	1.246	0.449	0.737	65.4	70.5
MGMT	1.148	0.802	1.643	0.450	0.737	69.5	66.5
LDHA	0.872	0.609	1.249	0.454	0.737	66.5	69.5
ITGB3	1.141	0.797	1.634	0.469	0.737	69.5	66.5
TYMS	0.891	0.622	1.274	0.526	0.764	63.8	72.3
CAV1	1.117	0.781	1.600	0.543	0.764	69.2	66.8
BCL2L11	1.115	0.779	1.595	0.552	0.764	72.7	63.2
PTEN	1.114	0.778	1.594	0.556	0.764	69.6	66.4
DPYD	1.106	0.773	1.583	0.581	0.771	67.7	68.3
PECAM1	1.102	0.770	1.577	0.596	0.771	68.7	67.3
BCL2	0.945	0.661	1.352	0.757	0.926	66.5	69.5
ERBB2	1.058	0.740	1.514	0.757	0.926	68.4	67.6
EGFR	0.968	0.677	1.385	0.861	0.973	65.6	70.4
GADD45A	1.030	0.720	1.474	0.871	0.973	66.8	69.1
HDAC1	1.027	0.718	1.469	0.884	0.973	70.2	65.8
THBS1	1.020	0.713	1.460	0.913	0.973	67.0	69.0
GGH	1.015	0.710	1.452	0.934	0.973	69.0	67.0
MLH1	1.010	0.706	1.444	0.958	0.973	69.8	66.1
BAX	1.007	0.704	1.441	0.968	0.973	68.4	67.6
RRM1	0.994	0.695	1.421	0.973	0.973	66.4	69.6
